# Pulmonary artery catheter monitoring versus arterial waveform-based monitoring during liver transplantation: a retrospective cohort study

**DOI:** 10.1038/s41598-023-46173-1

**Published:** 2023-11-15

**Authors:** Ji-Yoon Jung, Jin Young Sohn, Leerang Lim, Hyeyeon Cho, Jae-Woo Ju, Hyun-Kyu Yoon, Seong-Mi Yang, Ho-Jin Lee, Won Ho Kim

**Affiliations:** Department of Anesthesiology and Pain Medicine, Seoul National University Hospital, Seoul National University College of Medicine, 101 Daehak-Ro, Jongno-Gu, Seoul, 03080 Korea

**Keywords:** Medical research, Gastrointestinal diseases, Liver diseases

## Abstract

Although pulmonary artery catheter (PAC) has been used during liver transplantation surgery, the usefulness of PAC has rarely been investigated. We evaluated whether the use of PAC is associated with better clinical outcomes compared to arterial waveform-based monitoring after liver transplantation. A total of 1565 cases undergoing liver transplantation were reviewed. We determined whether patients received PAC or not and divided our cohort into the PAC with hemodynamic monitoring using PAC and the non-PAC with arterial waveform-based monitoring using FloTrac-Vigileo. Propensity score matching was performed. Acute kidney injury (AKI), early allograft dysfunction (EAD) and 1-year all-cause mortality or graft failure were compared in the matched cohorts. Logistic regression analysis was performed in the inverse probability of treatment-weighted (IPTW) cohort for postoperative EAD and AKI, respectively. Five-year overall survival was compared between the two groups. In the matched cohort, there was no significant difference in the incidence of AKI, EAD, length of hospital or ICU stay, and 1-year all-cause mortality between the groups. In the IPTW cohort, the use of PAC was not a significant predictor for AKI or EAD (AKI: odds ratio (95% confidence interval) of 1.20 (0.47–1.56), p = 0.229; EAD: 0.99 (0.38–1.14), p = 0.323). There was no significant difference in the survival between groups after propensity score matching (Log-rank test p = 0.578). In conclusion, posttransplant clinical outcomes were not significantly different between the groups with and without PAC. Anesthetic management without the use of PAC may be possible in low-risk patients during liver transplantation. The risk should be carefully assessed by considering MELD scores, ischemic time, surgical history, previous treatment of underlying liver disease, and degree of portal and pulmonary hypertension.

**Registration**: https://clinicaltrials.gov/ct2/show/NCT05457114 (registration date: July 15, 2022).

## Introduction

Pulmonary artery catheter (PAC) has been used for advanced hemodynamic monitoring during liver transplantation^1^. PAC is connected to a continuous cardiac output monitor (Vigilance II, Edward Lifesciences, Irvine, USA) and could provide continuous pulmonary artery pressure, and hemodynamic parameters of mixed venous oxygen saturation (SvO_2_), right ventricular end-diastolic volume, as well as continuous cardiac output. Baseline coagulopathy, portopulmonary hypertension, procedural difficulty of vascular anastomosis, clamping of inferior vena cava and graft reperfusion during liver transplantation frequently result in hemodynamic instability and massive bleeding, which necessitates advanced hemodynamic monitoring with PAC.

However, recent advances in arterial waveform-based monitoring can provide continuous cardiac output monitoring by arterial waveform analysis^2^. Using this technology, stroke volume variation is monitored as a preload index and its value was reported during liver transplantation^3^. Calculated systemic vascular resistance can be monitored if central venous pressure is provided. Although this arterial waveform-based hemodynamic monitoring may replace the PAC, PAC is still frequently used in United States academic centers^1^.

There have been several clinical trials that investigated the clinical impact of PAC by comparing the cases for whom PAC was not used^4,5^. They reported mixed results of no difference or increased mortality and end-organ complications in the group with PAC. Although these studies enrolled high-risk urgent or elective cardiac or non-cardiac surgery, clinical trials involving the patients undergoing liver transplantation have not been performed. Only small retrospective observational studies are found for liver transplantation^6,7^. These studies reported no association of PAC use with postoperative clinical outcomes including graft failure^3^. Another retrospective analysis reported that PAC monitoring combined with intraoperative transesophageal echocardiography (TEE) monitoring could reduce 30-day all-cause mortality. However, these studies were from a small sample size, and perioperative parameters that potentially affect mortality or graft failure were not fully adjusted in their statistical analysis.

Acute kidney injury (AKI) is one of the highly prevalent complications in the perioperative setting^8,9^. In patients undergoing living donor liver transplantation, the incidence of postoperative AKI ranged from 21 to 68%^10^. AKI after liver transplantation was associated with an increased 30-day mortality of 29% to 50%^11^, which further increases to 55 to 90% when renal replacement therapy is required^12^. High MELD score is a risk factor of AKI and the impact of AKI on graft and patient survival may be greater in patients with a high MELD score^13^.

Therefore, we attempted to add more robust evidence by performing a large retrospective cohort study. In this large retrospective observational study, we sought to compare the incidence of AKI and other clinical outcomes after liver transplantation between pulmonary artery catheter-based invasive monitoring and arterial waveform-based monitoring. We sought to evaluate whether the use of PAC is associated with better clinical outcomes compared to arterial waveform-based monitoring such as FloTrac Vigileo. For vigorous adjustment of confounding factors, we used the propensity score analysis.

## Methods

We obtained approval from the Seoul National University Hospital Institutional Review Board for this retrospective observational study (H-2205-117-1327). Our study was registered before data collection and statistical analysis on https://clinicaltrials.gov/ct2/show/NCT05457114 (registration date: July 15, 2022). We received a waiver of written informed consent from the Seoul National University Hospital Institutional Review Board, considering the retrospective nature of our study. All methods were performed following the relevant guidelines and regulations*.*

**Figure 1:**
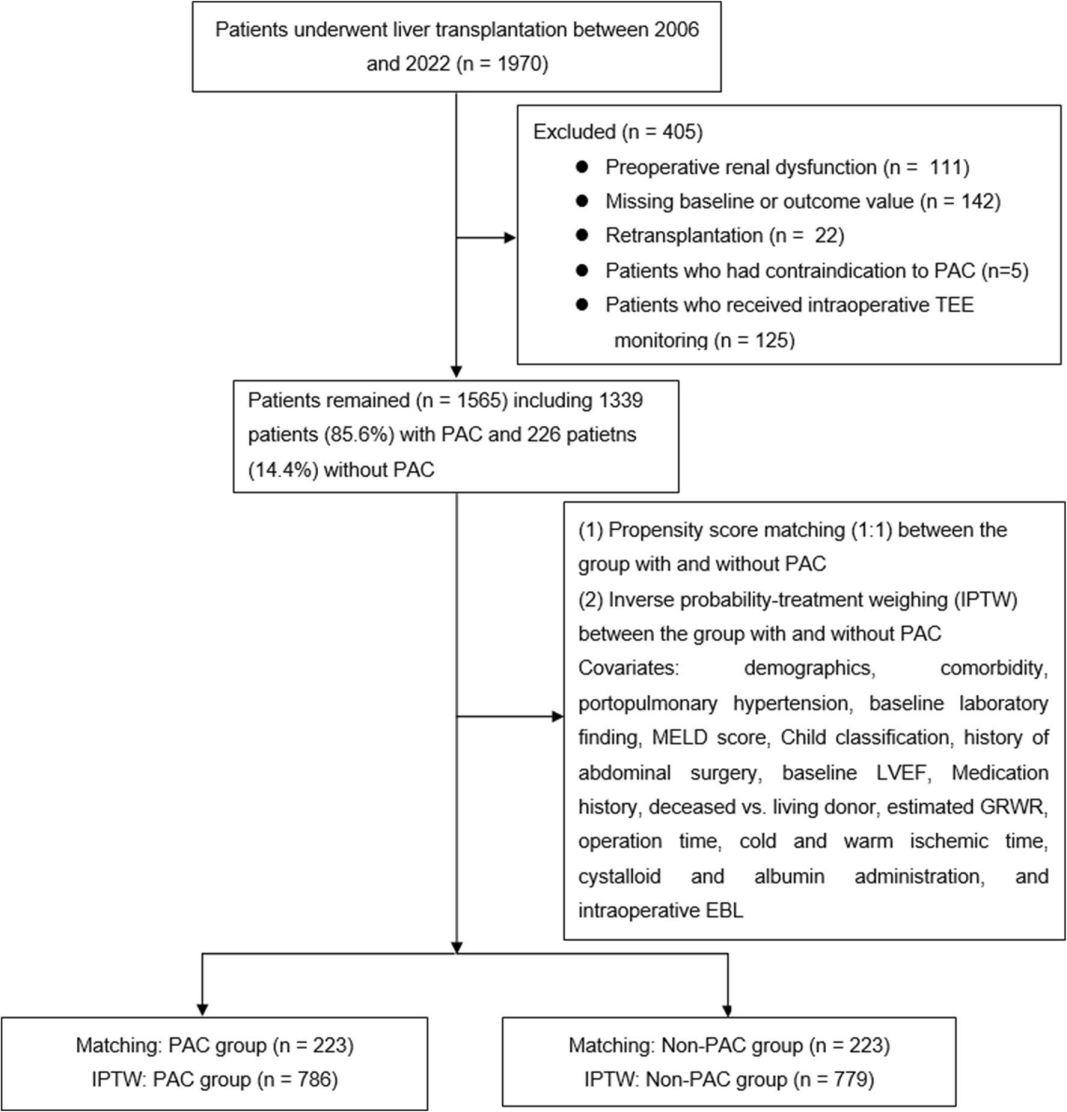
Flow diagram of data collection. *TEE* transesophageal echocardiography, *PAC* pulmonary artery catheter, *MELD* model for end-stage liver disease, *LVEF* left ventricular ejection fraction, *GRWR* graft recipient weight ratio, *EBL* estimated blood loss.

Our institutional electronic patient database of 1970 consecutive adult patients who underwent living or deceased donor liver transplantation at our tertiary care university hospital between 2006 and 2022 was reviewed. The patients with baseline renal dysfunction (n = 111), missing other baseline or outcome variables (n = 142), retransplantation (n = 22) and contraindication to PAC insertion (n = 5) were excluded. Those who received intraoperative TEE monitoring (n = 125) were also excluded because the patients without PAC were mixed with arterial waveform-based cardiac output monitoring with or without TEE (Fig. 1). The remaining 1565 patients were included in our analysis.

We extracted baseline characteristics or perioperative data reported to be associated with clinical outcomes including early allograft dysfunction (EAD) and acute kidney injury (AKI) after liver transplantation (Table 1)^13–18^. Postoperative clinical outcomes including the incidence of AKI^13,19,20^, early allograft dysfunction^18^, lengths of stay in intensive care unit (ICU), length of hospital stay, in-hospital all-cause mortality or graft failure, 1-year all-cause mortality or graft failure, and postoperative hemodialysis were collected.

**Table 1:** Comparison of patient characteristics and perioperative parameters between the group with and without pulmonary artery catheter (PAC).

Characteristic	PAC group (n = 1339)	Non-PAC group (n = 226)	P-value	Standardized difference
Demographic data				
Age, years	55 (49–61)	55 (50–61)	0.695	0.05
Female, n	373 (27.9)	72 (31.9)	0.217	0.09
Body-mass index, kg/m^2^	23.1 (21.1–25.3)	23.1 (21.0–25.5)	0.831	0.02
Background medical status				
Hypertension, n	135 (10.1)	24 (10.6)	0.805	0.02
Diabetes mellitus, n	208 (15.5)	45 (19.9)	0.098	0.11
Alcoholic liver cirrhosis, n	141 (10.5)	23 (10.2)	0.873	0.01
HBV hepatitis, n	553 (41.3)	97 (42.9)	0.647	0.03
HCV hepatitis, n	100 (7.5)	21 (9.3)	0.342	0.07
Hepatocellular carcinoma, n	569 (42.5)	85 (37.6)	0.169	0.10
Cholestatic disease, n	45 (3.4)	10 (4.4)	0.422	0.05
Preoperative hemoglobin, g/dl	10.9 (9.3–12.6)	10.3 (8.8–12.1)	0.004	0.21
Preoperative serum albumin level, mg/dl	3.0 (2.6–3.5)	3.0 (2.5–3.4)	0.307	0.05
MELD score	15 (12–22)	15 (10–22)	0.488	0.10
CTP score	8 (6–10)	8 (6–10)	0.753	0.03
Child class, n (A/ B/ C)	491 (36.7)/ 469 (35.0)/ 379 (28.3)	85 (37.6)/ 72 (31.9)/ 69 (30.5)	0.624	0.02
Previous abdominal surgery, n	57 (4.3)	10 (4.4)	0.908	0.01
Preoperative LVEF, %	65 (62–69)	65 (63–69)	0.454	0.05
Portopulmonary hypertension, n	12 (0.9)	-	0.153	0.13
Preoperative beta-blocker, n	52 (3.9)	9 (4.0)	0.943	0.01
Preoperative diuretics, n	52 (3.9)	10 (4.4)	0.700	0.03
Donor/graft factors				
Living/deceased, n	959 (71.6)/ 380 (28.4)	151 (66.8)/ 75 (33.2)	0.141	0.10
ABO-incompatible transplantation, n	103 (7.7)	39 (17.3)	< 0.001	0.29
Estimated GRWR	1.20 (1.04–1.40)	1.22 (1.05–1.45)	0.073	0.12
Operation and anesthesia details				
Year of transplant			< 0.001	0.79
2005–2010	229 (17.1)	11 (4.9)		
2011–2015	606 (45.3)	44 (19.5)		
2016–2022	504 (37.6)	171 (75.7)		
Operation time, hour	6.9 (5.7–8.0)	6.5 (5.7–7.8)	0.163	0.06
Cold ischemic time, min	89 (68–230)	87 (63–240)	0.548	0.06
Warm ischemic time, min	30 (26–35)	30 (27–34)	0.728	0.01
Intraoperative mean blood glucose, mg/dl	163 (149–180)	161 (143–178)	0.086	0.14
Intraoperative crystalloid administration, ml	3600 (2500–5250)	3500 (2300–5000)	0.205	0.13
Intraoperative hydroxyethyl starch administration, ml	0 (0–500)	0 (0–500)	0.548	0.02
5% albumin, ml	0 (0–0)	0 (0–0)	0.091	0.11
20% albumin, ml	200 (0–300)	200 (0–300)	0.074	0.13
Bleeding and transfusion amount				
pRBC transfusion, units	6 (2–12)	6 (2–10)	0.281	0.12
FFP transfusion, units	6 (0–12)	5 (0–10)	0.052	0.16
Platelet concentrate, units	0 (0–6)	0 (0–6)	0.007	0.12
Blood loss per body weight, ml/kg	47 (23–101)	40 (22–87)	0.166	0.03

The values are expressed as the median [interquartile range] or number (%).

There was no missing variable in the parameters listed in this table.

*CTP score* Child-Turcotte-Pugh score, *LVEF* left ventricular ejection fraction, *GRWR* graft versus recipient body weight ratio, *p-RBC* packed red blood cells, *FFP* fresh frozen plasma.

The primary outcome of our study was AKI diagnosed by the KDIGO criteria, which was defined according to the greatest change in serum creatinine level during the postoperative seven days (Stage 1: more than 1.5-fold increase from baseline or absolute increase in serum creatinine of ≥ 0.3 mg/dL; stage 2: more than twofold; stage 3: more than threefold increase from baseline level or increase in serum creatinine to ≥ 4.0 mg/dL or the initiation of renal replacement therapy)^13,19,20^. The most recent serum creatinine level measured before surgery was collected as a baseline value.

Early allograft dysfunction was defined when one or more of the following are present: total bilirubin ≥ 10 mg/dL, prothrombin time: international normalized ratio ≥ 1.6 on the seventh postoperative day, or aspartate or alanine transaminase > 2000 IU/L within the first 7 postoperative days^18^. Surgical complication rates including graft dysfunctions, vascular complications, biliary complications, and wound infection were compared by Clavien-Dindo classification^21^. PAC insertion-related complications including ventricular arrhythmia, right bundle branch block, complete heart block, guidewire embolism, catheter knotting, and failure to insert into the pulmonary artery were collected.

The primary exposure variable of our interest was the PAC (Edward Lifesciences, Irvine, California, USA) insertion for hemodynamic monitoring during liver transplantation surgery. PAC was connected to a continuous cardiac output monitor (Vigilance I from 2006 to 2012; Vigilance II from 2012 to 2022, Edward Lifesciences, Irvine, USA). Continuous pulmonary artery pressure, SvO_2_, right ventricular end-diastolic volume, and continuous cardiac output were monitored for hemodynamic management. A decision to insert PAC was made individually at the discretion of attending anesthesiologists during the study period. PAC was not inserted simply because the patient had a high Models for End-stage Liver Disease (MELD) score. PAC was not inserted for the following contraindications of PAC insertion—right-sided endocarditis, right heart mass, tricuspid or pulmonic valvular stenosis or vegetation or other pathology and under the following circumstances—(1) development of severe arrhythmia or repeated kinking of the catheter, or (2) failure of the catheter to proceed to the pulmonary artery. The patients who had these contraindications were excluded from our analysis because the cardiac conditions could affect the clinical outcomes after liver transplantation.

For our statistical analysis, we divided our retrospective cohort into PAC and non-PAC groups. In the PAC group, PAC was inserted, and PAC-derived hemodynamic parameters were monitored. In the non-PAC group, arterial waveform-based monitoring with FloTrac Vigileo system (EV1000 clinical platform, Edward Lifesciences, Irvine, California, USA) was performed for continuous cardiac output monitoring^22,23^. Stroke volume, stroke volume variation, and systemic vascular resistance were also monitored by the FloTrac Vigileo system. If intraoperative TEE was monitored, the case was excluded from our analysis because we attempted to compare the hemodynamic management according to the hemodynamic parameters by PAC and arterial waveform analysis.

### Statistical analysis

We assessed the normality of our continuous data using the Shapiro–Wilk test. Continuous data were presented as the median (25 and 75 percentiles) and incidence data were reported as numbers (%). Continuous data were compared between groups by the Mann–Whitney *U* test and incidence data were compared by or chi-square test or Fisher’s exact test depending on their expected counts. Baseline or outcome data were missing in 7.4% of records. We excluded these missing cases before our analysis. The missing was considered random, and we could not find any significant difference in the available baseline characteristics between the excluded and included cases in our preliminary analysis.

The following is the summary of our main analysis. All analyses were performed based on an a priori statistical analysis plan unless they were indicated as post hoc analysis. We published our prespecified statistical analysis plan on the following internet site (https://doi.org/10.6084/m9.figshare.20334984.v1) (Supplemental Text S1). During our analysis process, we determined to omit the binary multivariable logistic regression analysis to evaluate the association of the use of PAC with the risk of EAD and AKI because there are too many analytic methods in our plan which increases the type 1 error by multiple testing.

Firstly, a propensity-score matching analysis was conducted to further adjust the possible confounding effect of our independent parameters of the baseline characteristics and surgical and anesthesia-associated parameters^24^. Using non-parsimonious logistic regression modeling, we calculated propensity scores for the use of PAC vs. no use of PAC including the following covariates for matching: sex, age, body-mass index, history of diabetes mellitus, hypertension, portopulmonary hypertension, baseline hemoglobin level, preoperative serum albumin level, MELD score, Child classification, Child-Turcotte-Pugh score, history of previous abdominal surgery, baseline left ventricular ejection fraction, preoperative beta-blocker, diuretics administration, deceased or living donor, estimated graft-recipient body-weight ratio, year of transplantation, operation time, cold ischemic time (CIT) and warm ischemic time (WIT), intraoperative mean blood glucose, the amount of intraoperative hydroxyethyl starch or albumin administration, the amount of intraoperative transfusion, intraoperative estimated blood loss. These covariates were selected according to the previous studies reporting the risk factors of AKI^13–16,25,26^ or EAD^27,28^. We set the caliper width as 0.2 standard deviations of the logit-transformed propensity score. The ratio of matching between the PAC and non-PAC was 1:1 and 240 paired sets of patients were generated. We defined that baseline variables are different if the absolute standardized difference > 0.304^29^. Then we compared the incidence of intraoperative events including postreperfusion syndrome (PRS)^30^ and postoperative clinical outcomes including EAD, AKI between the matched groups.

Secondly, for another propensity score analysis, we performed a multivariable logistic regression analysis using inverse probability of treatment-weighting (IPTW) for postoperative EAD and AKI, separately^24,31^. This was to evaluate the robustness of our propensity score matching analysis and to avoid the power reduction by propensity score matching. During propensity score matching, a substantial number of cases are excluded due to the inability to find an appropriate match which limits the generalizability and interpretability of analysis results. IPTW incorporates the entire study population and weights the cases according to the inverse of the probability of receiving the use of PAC in order to generate a balanced pseudo population. It is also easy to show balance on baseline parameters after weighting. We used Brant’s Wald test and the likelihood ratio test to investigate the proportional odds assumption. To meet the positivity assumption of IPTW analysis, we excluded the cases with a probability of receiving PAC of 1 or 0 from our analyses. Additionally, we replaced extreme weights greater than the 99th percentile or less than the lowest first with the value of 99th or the first percentile, by weight trimming.

Thirdly, Kaplan–Meier survival curve analysis for the overall or graft survival was conducted between the non-PAC and PAC during the first postoperative year. We used the log-rank test to evaluate the survival difference between the two groups.

Fourthly, as a post-hoc analysis to address the time-dependent bias due to the long period of our cohort, we analyzed the subgroup of our retrospective cohort from the year 2016 to 2022 (n = 675). Multivariable logistic regression analysis and propensity score analyses including matching and IPTW analysis were performed in this subgroup. The year 2016 to 2022 was chosen because 75.7% of the non-PAC cases were in this period. Before the year 2016, the number of cases managed without a PAC was very small and both strategies were consistently employed only after the year 2016.

Finally, to address whether the association of PAC monitoring with clinical outcomes is different across to the different risks of patients, we performed stratified subgroup analyses according to MELD score, WIT and CIT. We stratified the high and low-risk subgroups according to the cutoffs of the highest quartile and lower three quartiles of MELD, WIT and CIT. Then we compared the incidence of EAD, AKI and other clinical outcomes between the groups.

All P values are calculated for two-tailed hypothesis testing, and the significance level of 0.005 was used for statistical significance given the multiple testing for our secondary outcomes. We used Stata 15.1 (StataCorp, College Station, TX, USA) for statistical analyses and Medcalc Statistical Software version 18.6 (MedCalc Software bvba, Ostend, Belgium) to draw the Kaplan–Meier survival curve.

## Results

Among our dataset of 1565 liver transplant cases, 1339 patients received PAC insertion and hemodynamic monitoring by PAC-derived parameters. The remaining 226 patients did not receive PAC insertion and were monitored with FloTrac Vigileo (Fig. 1).

Table 1 shows the baseline characteristics and perioperative variables between the PAC and non-PAC. We found significant differences in age, comorbidities, the severity of liver cirrhosis, MELD score, preoperative medication, year of transplantation, surgery, and anesthesia-related parameters.

The overall incidence of AKI was 42.4% (n = 664) and that of EAD was 2.9% (n = 45). Supplemental Table S1 compares the baseline and perioperative parameters before and after propensity score matching. Supplemental Figure S1 compares the distribution of propensity scores and the standard difference before and after propensity score matching. Table 2 shows the comparison of clinical outcomes including EAD and AKI before and after propensity score matching. There was no significant difference in the incidence of AKI between the PAC (n = 198/473, 41.9%) and non-PAC (n = 328/744, 44.1%, p = 0.445) after matching. The length of hospital or ICU stay was not significantly different after propensity score matching.

**Table 2:** Comparisons of intraoperative event and secondary clinical outcomes after liver transplantation between the PAC and non-PAC group before and after propensity score matching.

	Before propensity score matching			After propensity score matching		
	PAC group (n = 1339)	Non-PAC group (n = 226)	P-value	PAC group (n = 223)	Non-PAC group (n = 223)	P-value
Intraoperative events						
Transfusion ≥ 10 units of pRBC	202 (15.1)	35 (15.5)	0.876	35 (15.7)	35 (15.7)	0.999
Postreperfusion syndrome, n	318 (23.7)	58 (25.7)	0.533	56 (25.1)	51 (22.9)	0.579
Epinephrine infusion > 0.05 μg/kg/min	32 (2.4)	5 (2.2)	0.871	4 (1.8)	5 (2.2)	0.736
Norepinephrine infusion > 0.20 μg/kg/min	110 (8.2)	19 (8.4)	0.923	18 (8.1)	17 (7.6)	0.860
Postoperative outcomes						
Acute kidney injury, n			0.295			0.960
Stage 1, n	422 (31.5)	57 (25.2)		62 (27.8)	57 (25.6)	
Stage 2, n	97 (7.2)	18 (8.0)		18 (8.1)	18 (8.1)	
Stage 3, n	60 (4.5)	10 (4.4)		9 (4.0)	9 (4.0)	
Acute kidney injury, all stage, n	579 (43.2)	85 (37.6)	0.113	89 (39.9)	84 (37.7)	0.627
Postoperative hemodialysis, n	91 (6.8)	17 (7.5)	0.690	13 (5.8)	16 (7.2)	0.565
Postoperative bleeding, n	58 (4.3)	9 (4.0)	0.810	13 (5.8)	9 (4.0)	0.382
Postoperative wound infection, n	39 (2.9)	3 (1.3)	0.173	7 (3.1)	3 (1.3)	0.201
Early allograft dysfunction, n	38 (2.8)	7 (3.1)	0.829	6 (2.7)	7 (2.7)	0.778
In-hospital mortality, n	17 (1.3)	2 (0.9)	0.625	4 (1.8)	2 (0.9)	0.411
1-year mortality, n	64 (4.8)	9 (4.0)	0.599	10 (4.5)	9 (4.0)	0.815
Length of ICU stay, days	5 (4–7)	5 (4–8)	0.990	5 (4–7)	5 (4–8)	0.921
Length of hospital stay, days	19 (15–27)	19 (15–29)	0.410	18 (15–31)	19 (15–29)	0.669

Data are presented as the number (%) or median [interquartile range] or number (%).

Postreperfusion syndrome was defined as a > 30% decrease of mean blood pressure at least 1 min within 5 min of portal vein reperfusion compared with the baseline observed immediately before reperfusion.

*ICU* intensive care unit.

IPTW yielded the cohort of PAC (n = 786) and non-PAC (n = 779). Supplemental Table S2 shows the comparison of patient characteristics and perioperative parameters between the groups with and without PAC in an inverse probability of treatment-weighted cohort. Ordinal multivariable logistic regression analysis in the inverse probability of treatment-weighted cohort (n = 1565) revealed that the use of PAC was not found to be associated with EAD (OR 0.99, 95% CI 0.38–1.14, p = 0.323) or AKI (OR 1.20, 95% CI 0.47–1.56, p = 0.229).

**Figure 2:**
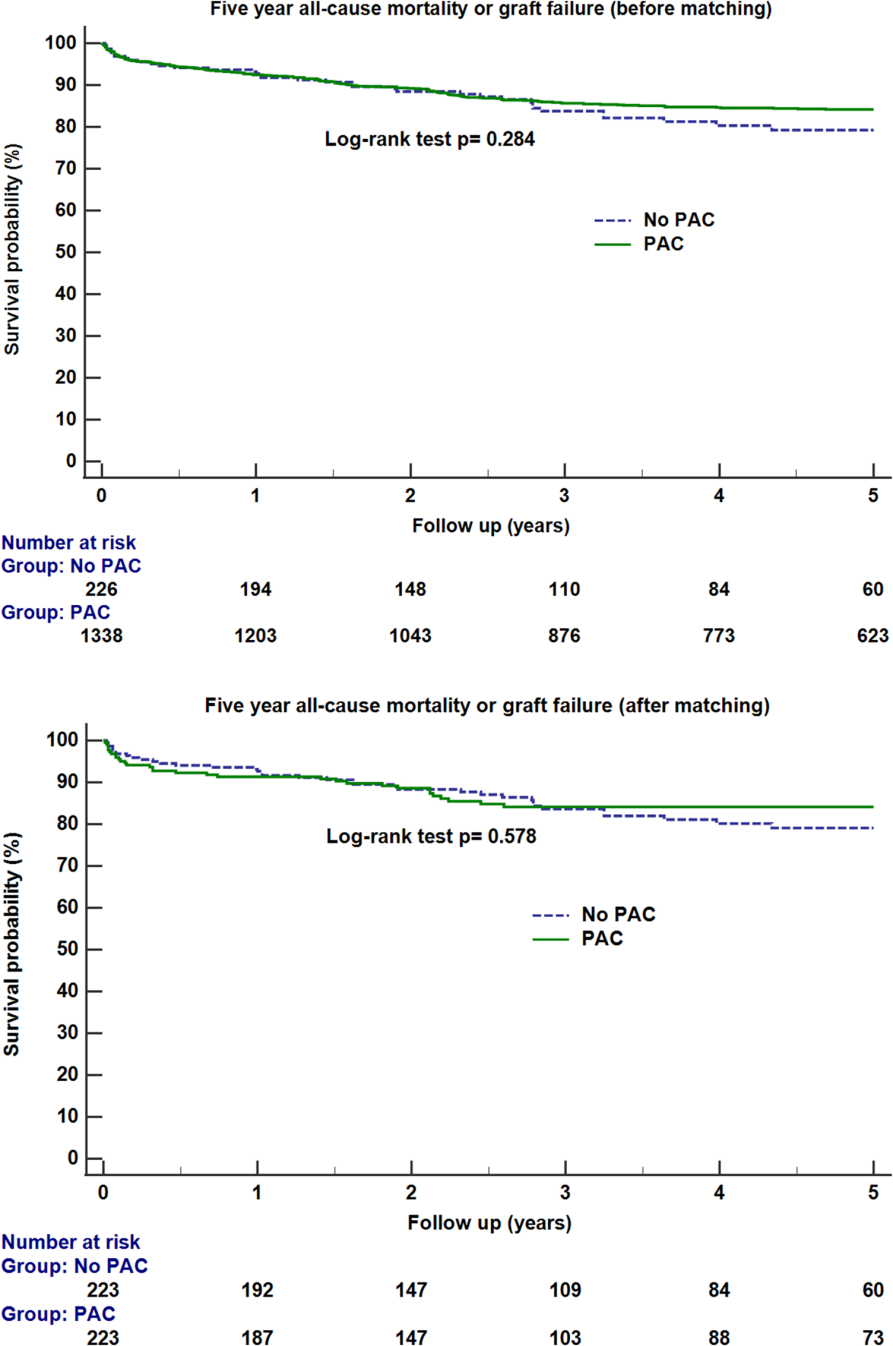
Kaplan–Meier survival curve analysis between the PAC and non-PAC groups before and after propensity score matching. The results of the log-rank test between the groups are shown in the figure. The number of patients who had follow-up data at each time point is shown below the figure. *PAC* pulmonary artery catheter.

The results of Kaplan–Meier survival curve analyses before and after propensity score matching between the PAC and non-PAC are shown in Fig. 2. Log-rank test shows no significant difference in the survival between the two groups (p = 0.284 and p = 0.578 before and after matching, respectively).

Supplemental Table S3 compares the baseline characteristics between PAC and non-PAC in the subgroup of the patients who underwent surgery after the year 2016 (n = 675). Propensity score matching analysis yielded 166 pairs of patients and the comparisons of secondary clinical outcomes are shown in Supplemental Table S4. We could not find any significant difference in these outcomes. The results of logistic regression analysis in the inverse probability of treatment-weighted cohort (n = 718) are shown in Supplemental Table S5. The use of PAC did not make any significant difference.

Supplemental Tables S6 and S7 show the results of the stratified subgroup analysis of high and low MELD score. In the high MELD score group, there was a significant difference in the postreperfusion syndrome between PAC and non-PAC groups after matching. Supplemental Tables S8 and S9 show the analysis for high and low CIT groups. In the high CIT group, the incidences of postreperfusion syndrome and postoperative AKI were significantly different between PAC and non-PAC groups. Supplement Tables S10 and S11 report the analysis for high and low WIT groups. There was no significant difference between groups.

## Discussion

In patients undergoing liver transplantation, there was no significant difference in our primary outcome of AKI as well as all other secondary outcomes including EAD and 1-year all-cause mortality or graft failure regardless of the use of PAC monitoring. To adjust possible confounding factors of baseline characteristics and perioperative parameters that potentially affect the risk of clinical outcomes, we performed propensity score matching and logistic regression analysis in the inverse probability treatment-weighted cohort. Our results suggest that PAC monitoring does not affect clinical outcomes including graft function. However, our stratified subgroups analysis for high and low MELD scores and ischemic times showed significant differences in the incidence of PRS and AKI in the subgroup of high MELD scores or high CIT. Therefore, hemodynamic management during liver transplantation without the use of PAC may be possible for patients with low MELD scores and short ischemic time.

Liver transplantation requires a long operation time and is often associated with a significant amount of surgical bleeding. It is common for the anesthesiologist to infuse large amounts of fluid or blood products due to massive bleeding, hemodynamic instability, or ascites drainage.

Therefore, in the anesthesia for liver transplantation, careful and detailed hemodynamic management is required and advanced hemodynamic monitoring such as cardiac output and SvO_2_ has been used. Among the PAC-derived hemodynamic parameters, right ventricle end-diastolic volume and SvO_2_ are monitored to guide intravascular volume status. Although FloTrac Vigileo monitoring provides continuous cardiac output and stroke volume variation as an indicator of fluid responsiveness and preload, some may argue that SvO_2_ and cardiac output measured by thermodilution technique would be more accurate compared to the arterial waveform analysis.

Previous clinical trials that investigated the impact of the use of PAC on the clinical outcomes enrolled patients undergoing high-risk urgent or elective surgery other than liver transplantation^4^. They found no clinical benefit from using PAC and only the incidence of pulmonary thromboembolism was significantly higher in the group with PAC^4^. Another clinical trial for the patients undergoing coronary artery bypass reported increased mortality and end-organ complications in the group with PAC^5^.

However, there are only small retrospective observational studies for liver transplantation^6,7^. The use of PAC compared to the arterial waveform-based monitoring of FloTrac Vigileo was not associated with any difference in the duration of postoperative ventilator care, the incidence of graft failure, or length of hospital stay^6^. Nonetheless, the limitation of FloTrac Vigileo should be acknowledged. Arterial pulse waveform analysis by FloTrac Vigileo could be incorrect under severe vasoconstriction by use of vasopressors in these cases with massive bleeding and low temperature. Another retrospective study compared clinical outcomes between one group with PAC, another group with TEE monitoring, and the other group with both PAC and TEE monitoring. The length of hospital stay was shorter, and 30-day all-cause mortality was significantly lower in the group with both PAC and TEE monitoring. However, these studies were with small sample size and adjustment for the confounding parameters was not sufficient.

Furthermore, TEE gained growing popularity for intraoperative hemodynamic monitoring during liver transplantation^32,33^. Intraoperative TEE could provide the status of cardiac contractility, wall motion abnormality, and preload as well as elevated right heart pressure. Cardiac output can be calculated by Doppler measurements. As intraoperative TEE can also detect catastrophic hemodynamic events such as air embolism and pulmonary thromboembolism in addition to left ventricular outflow tract obstruction, TEE could replace PAC-derived hemodynamic monitoring. However, as learning to perform TEE takes time^34^, the skills necessary to use TEE should be well acquired before arterial waveform-based monitoring by FloTrac Vigileo can be considered in the selective low-risk patients and TEE could be a backup strategy for arterial waveform-based monitoring.

Although our institution started intraoperative TEE monitoring about a year ago, we could not compare TEE monitoring and PAC use because most of the intraoperative TEE monitoring was used in combination with PAC use in our cohort. We excluded the patients with TEE monitoring to compare the pure association of PAC with that of arterial waveform-based monitoring.

Recently, expert panel recommendations were published regarding optimal anesthetic monitoring during liver transplantation^35^. Although the statements were based on small retrospective observational studies, they strongly recommended PAC monitoring, particularly in cases with portopulmonary hypertension, hemodynamic instability, and cardiac dysfunction. However, as demonstrated by our study results, PAC monitoring may not be necessary for elective liver transplantation cases without portopulmonary hypertension or cardiac dysfunction associated with cirrhotic cardiomyopathy. Although hemodynamic instability develops during graft reperfusion, it is often transient and only a small dose of vasopressor or epinephrine is sufficient even for the case of PRS.

We performed stratified analyses to evaluate whether the association of PAC monitoring with clinical outcomes differs according to the baseline risk of the patients in terms of MELD score, CIT and WIT. Compared to low MELD patients, high MELD patients tend to already have renal insufficiency or severe coagulopathy, which makes high MELD patients more vulnerable to hemodynamic instability, fluid shifting, and metabolic acidosis^36^. Therefore, a necessity for advanced hemodynamic monitoring could be different according to MELD score. We found several significant differences in the incidence of PRS and AKI in the subgroup of high MELD score or high CIT, which may mean that PAC monitoring could make a difference in the clinical outcomes compared to non-PAC monitoring. Arterial waveform-based monitoring could be suggested only to the patients with low-risk patients with low MELD scores or low CIT.

Our study has several important limitations. Firstly, although we performed propensity score analysis with rigorous adjustment of covariates, our study was a single-center retrospective observational study. Propensity score matching and IPTW only reduce but do not exclude the selection bias. An unmeasured or unknown confounding factor may affect our analysis results. Some may have concerns that non-PAC monitoring was chosen only in easier cases and the patients who received TEE monitoring were excluded due to the complexity of cases. However, we excluded the patients with TEE monitoring to compare PAC with Flo-Trac Vigileo monitoring, and TEE was used as a full-time monitoring from anesthesia induction, not as a rescue monitoring. Secondly, the external validity of our analysis is limited. In other institutions where the proportion of patients with advanced hepatic failure is high, the requirements for monitoring pulmonary artery pressure would be greater compared to our institution where most of the cases are living donor transplantation with lower MELD scores. Thirdly, the association of recently increasing intraoperative TEE monitoring with our outcomes was not investigated in our study. Further studies are required to investigate whether TEE monitoring could substitute the use of PAC. Fourthly, our cohort was from a relatively long period. Although we analyzed the timing of surgery as a covariate in our multivariable analysis and propensity analysis, the versions of cardiac output monitoring for PAC and FloTrac Vigileo have been updated during the data collection period. The update of software may have reduced the difference in the accuracy of cardiac output measurement between the thermodilution technique and arterial waveform analysis. The surgical technique, the amount of bleeding and transfusion have improved over time which could be a confounding factor in interpreting our analysis results. To address this, we analyzed the subgroup of recent years, but we could not find any significant results. Fifthly, the incidence of postreperfusion syndrome was not compared between groups. As the severity of baseline liver disease increases, monitoring with PAC would be more valuable and may yield significant differences in the clinical outcomes. Sixthly, we defined postoperative AKI based on KDIGO criteria, using serum creatinine changes only. Urine output criteria were not used in our analysis. Finally, new definition of EAD such as the Liver Graft Assessment Following Transplantation (L-GrAFT) score and the Early Allograft Failure Simplified Estimation (EASE) score has been introduced^37–39^. Further studies should consider these new definitions of EAD.

In conclusion, in this large single-center retrospective observational study, we could not find any significant difference in clinical outcomes after liver transplantation between the group that used PAC and the group without PAC. Based on our results, anesthesia without the use of PAC during liver transplantation may be possible for low-risk patients. However, our study results should be interpreted cautiously because our cohort included many elective living donor liver transplants with low MELD scores, short ischemic time, and a low incidence of pulmonary hypertension. Our subgroup analysis revealed a significant difference in the incidence of PRS between the PAC group and the non-PAC group in the subgroup with a high MELD score. Also, in the high CIT group, both PRS and AKI showed significant differences between groups. These results suggest that it is important to delicately identify candidates to apply arterial waveform-based monitoring instead of PAC monitoring. The risk should be carefully assessed by considering MELD scores, ischemic time, surgical history, previous treatment of underlying liver disease, and degree of portal and pulmonary hypertension. Cardiac output monitoring with arterial waveform analysis may replace the use of PAC during liver transplantation. However, even for low-risk patients, a back-up hemodynamic strategy such as TEE should be prepared.

### Supplementary Information

Supplementary Information.

## Data Availability

The datasets generated during and/or analyzed during the current study are available from the corresponding author on reasonable request.

## Abbreviations

AKI: Acute kidney injury
CIT: Cold ischemic time
EAD: Early allograft dysfunction
IPTW: Inverse probability of treatment-weighting
MELD: Model for end-stage liver disease
PAC: Pulmonary artery catheter
PRS: Postreperfusion syndrome
SvO_2_: Mixed venous oxygen saturation
WIT: Warm ischemic time
TEE: Transesophageal echocardiography
