# Supplementary material for: Pulmonary artery catheter monitoring versus arterial waveform-based monitoring during liver transplantation: a retrospective cohort study
Source: Sci Rep. 2023 Nov 15;13:19947. doi: 10.1038/s41598-023-46173-1 (PMC10651933; doi:10.1038/s41598-023-46173-1)
Supplement: Supplementary file 1 — Supplementary Information. [file 41598_2023_46173_MOESM1_ESM.docx]

**Pulmonary artery catheter moniotoring versus arterial waveform-based monitoring during liver transplantation: a retrospective cohort study**

: **Supplemental Materials**

**Supplemental Text S1.** Statistical analysis plan.

**Supplemental Table S1**. Comparison of patient characteristics and perioperative parameters between the group with and without pulmonary artery catheter (PAC) after propensity score matching.

**Supplemental Table S2**. Comparison of patient characteristics and perioperative parameters between the group with and without pulmonary artery catheter (PAC) in the inverse probability of treatment weighted cohort.

**Supplemental Table S3**. Comparison of patient characteristics and perioperative parameters between the group with and without pulmonary artery catheter (PAC) in the subgroup of the patients who underwent surgery after the year 2016.

**Supplemental Table S4**. Comparisons of secondary clinical outcomes after liver transplantation between the PAC and non-PAC group before and after propensity score matching in the subgroup of the patients who underwent surgery after the year 2016.

**Supplemental Table S5**. Results of ordinal logistic regression analysis for early allograft dysfunction and acute kidney injury after liver transplantation using inverse probability of treatment weighting in the subgroup of the patients who underwent surgery after the year 2016.

**Supplemental Table S6**. Subgroup analysis of the highest quartile of model for end-stage liver disease score (≥ 22) for the comparisons of intraoperative event and secondary clinical outcomes after liver transplantation between the PAC and non-PAC group before and after propensity score matching (n=398).

**Supplemental Table S7**. Subgroup analysis of the low three quartiles of model for end-stage liver disease score (< 22) for the comparisons of intraoperative event and secondary clinical outcomes after liver transplantation between the PAC and non-PAC group before and after propensity score matching (n = 1167).

**Supplemental Table S8**. Subgroup analysis of the highest quartile of cold ischemic time (≥ 240 min) for the comparisons of intraoperative event and secondary clinical outcomes after liver transplantation between the PAC and non-PAC group before and after propensity score matching (n = 454).

**Supplemental Table S9**. Subgroup analysis of the low three quartiles of cold ischemic time (< 240 min) for the comparisons of intraoperative event and secondary clinical outcomes after liver transplantation between the PAC and non-PAC group before and after propensity score matching (n = 1111).

**Supplemental Table S10**. Subgroup analysis of the highest quartile of warm ischemic time (≥ 35 min) for the comparisons of intraoperative event and secondary clinical outcomes after liver transplantation between the PAC and non-PAC group before and after propensity score matching (n = 425).

**Supplemental Table S11**. Subgroup analysis of the low three quartiles of warm ischemic time (< 35 min) for the comparisons of intraoperative event and secondary clinical outcomes after liver transplantation between the PAC and non-PAC group before and after propensity score matching (n = 1140).

**Supplemental Figure S1**. Histogram (left) shows the distribution of propensity scores before and after matching. Covariate balance plot (right) compares the standardized differences before and after the propensity score matching.

**Supplemental Text S1.** Statistical analysis plan.

**1. Definition of the primary exposure variable**

The primary exposure variable of our interest will be the pulmonary artery catheter or Swan-Ganz catheter (Edward Lifesciences, Irvine, Califonia, USA) insertion for hemodynamic monitoring during liver transplantation surgery.

A pulmonary artery catheter (PAC) was connected to a continuous cardiac output monitor (Vigilance I from 2006 to 2012; Vigilance II from 2012 to 2022, Edward Lifesciences, Irvine, USA). Continuous pulmonary artery pressure, mixed venous oxygen saturation (Svo2), right ventricular end-diastolic volume, and continuous cardiac output was monitored for hemodynamic management.

A decision to insert PAC was made individually at the discretion of attending anesthesiologists during the study period. PAC was not inserted simply because the patient had a high MELD score.

PAC was not inserted for the following contraindications of PAC insertion - right-sided endocarditis, right heart mass, tricuspid or pulmonic valvular stenosis or vegetation or other pathology and under the following circumstances – 1) the patient already had central vein catheter preoperatively, 2) development of severe arrhythmia or repeated kinking of the catheter, or 3) failure of the catheter to proceed to the pulmonary artery.

As a control group, we will categorize our retrospective cohort into two groups of the PAC group and non-PAC group. In the PAC group, PAC was inserted and PAC-derived hemodynamic parameters were monitored. In the non-PAC group, less invasive monitoring with FloTrac Vigileo system (EV1000 clinical platform, Edward Lifesciences, Irvine, California, USA) was performed for continuous cardiac output monitoring.^1,2^ Stroke volume, stroke volume variation, and systemic vascular resistance were also monitored by the FloTrac Vigileo system. If intraoperative transesophageal echocardiography was monitored, the case will be excluded from our analysis because we attempt to compare the hemodynamic management according to the hemodynamic parameters by PAC and less-invasive arterial waveform analysis.

**2. Definition of primary clinical outcomes**

Primary outcome:

acute kidney injury (AKI):

We will define AKI by the KDIGO criteria, which will be determined according to the greatest change in serum creatinine level during the postoperative seven days (Stage 1: more than 1.5-fold; stage 2: more than 2-fold; stage 3: more than 3-fold increase of baseline or increase in SCr to ≥ 4.0 mg/dL or the initiation of renal replacement therapy).^3-5^

The most recent SCr level measured before surgery will be collected as a baseline value.

**3. Definition of secondary clinical outcomes**

Postoperative outcome parameters including the incidence of early allograft dysfunction,^6^ lengths of intensive care unit stay, length of hospital stay, in-hospital all-cause mortality or graft failure, one-year all-cause mortality or graft failure, and postoperative hemodialysis the incidence of AKI, ^3-5^ early allograft dysfunction,^6^ lengths of intensive care unit stay, length of hospital stay, in-hospital all-cause mortality or graft failure, one-year all-cause mortality or graft failure, and postoperative hemodialysis will be collected.

Early allograft dysfunction will be defined when one or more of the following are present within the first 7 postoperative days: total bilirubin ≥ 10 mg/dL, prothrombin time: international ratio ≥ 1.6, or aspartate transaminase/alanine transaminase > 2000 IU/L.^6^

**4. Determination of normality, data presentation, handling of missing values and comparison of baseline characteristics**

We will determine the normality of our continuous data using the Shapiro-Wilk test. Continuous data will be presented as the median (25 and 75 percentiles) and incidence data as the number (%). Continuous data will be compared between groups by the Mann-Whitney U test and incidence data will be compared by or χ^2^ test or Fisher’s exact test depending on their expected counts.

If missing data of baseline or outcome data are less than 10% of records, we will compare the available data between the cases with and without missing values. If there are no significant differences between the baseline or perioperative parameters, we will exclude these missing cases before our analysis.

If missing data of baseline or outcome data are more than 10% of records, we will impute the missing data with multiple imputations with ordinal logistic regression, assuming missing at random. The imputation model will include all independent variables plus the outcome variables of our analysis.

**5. Multivariable logistic regression analysis for AKI**

We will perform a multivariable logistic regression analysis to evaluate the association of the use of PAC with the risk of EAD and AKI. Two separate regression analyses will be performed for EAD and AKI, respectively.

For the primary exposure variable, we will use the binomial variable of the use of PAC versus the use of FloTrac. Covariates of baseline characteristics or perioperative data will be included in the regression analysis. No variable selection process will be used in the regression analysis. We will assess the calibration and discrimination of our regression model by the Hosmer-Lemeshow goodness of fit test and c-statistics, respectively.

**5. Propensity score analysis – matching**

A propensity-score matching analysis will be conducted to adjust the possible confounding effect of our independent parameters of the baseline characteristics and surgical and anesthesia-related parameters.^7^

Using non-parsimonious logistic regression modeling, we will calculate propensity scores for the use of PAC vs. no use of PAC including the following covariates for matching:

(1) Demographics: sex, age, body mass index

(2) Past medical history & comorbidity: history of diabetes mellitus, hypertension, portopulmonary hypertension, history of previous abdominal surgery

(3) Baseline liver disease severity & laboratory findings: baseline hemoglobin level, preoperative serum albumin level, Models for end-stage liver disease (MELD) score, Child classification, Child-Turcotte-Pugh (CTP) score, baseline left ventricular ejection fraction

(4) Preoperative medication: preoperative beta-blocker, diuretics administration,

(5) Donor-related parameters: deceased or living donor, estimated graft-recipient body-weight ratio

(6) Surgery: year of transplantation, operation time, cold and warm ischemic time, intraoperative mean blood glucose, the amount of intraoperative hydroxyethyl starch or albumin administration, the amount of intraoperative transfusion, intraoperative estimated blood loss.

We will set the caliper width as 0.2 standard deviations of the logit-transformed propensity score. The ratio of matching between the PAC and non-PAC groups was 1:1 and 240 paired sets of patients were generated. Then we compared the incidence of EAD, AKI and other clinical outcomes between the matched groups.

**7. Propensity score analysis - Inverse probability of treatment weighting (IPTW)**

For another propensity score analysis, we will perform a multivariable logistic regression analysis using inverse probability of treatment-weighting (IPTW) for postoperative EAD and AKI, separately.^8,9^ This is to evaluate the robustness of our propensity score matching analysis and to avoid the power reduction by propensity score matching.

During propensity score matching, a substantial number of cases are excluded due to the inability to find an appropriate match which limits the generalizability and interpretability of analysis results. IPTW incorporates the entire study population and weights the cases according to the inverse of the probability to receive the use of PAC in order to generate a balanced pseudo population.

We plan to use Brant’s Wald test and the likelihood ratio test to investigate the proportional odds assumption. To meet the positivity assumption of IPTW analysis, we may exclude the cases with a probability to receive PAC of 1 or 0 from our analyses. Additionally, we will replace extreme weights greater than the 99th percentile or less than the lowest first with the value of 99th or the first percentile, by weight trimming.

**8. Kaplan-Meier survival curve analysis**

Kaplan-Meier survival curve analysis for the overall or the graft survival will be conducted between the non-PAC and PAC group during the first postoperative year. We will use the log-rank test to evaluate the survival difference between the two groups.

**9. Statistical software**

All P values will be calculated for two-tailed hypothesis testing, and the significance level of 0.05 will be used for statistical significance.

We will use Stata 15.1 (StataCorp, College Station, TX, USA) for statistical analyses and Medcalc Statistical Software version 18.6 (MedCalc Software bvba, Ostend, Belgium) to draw the Kaplan-Meier survival curve.

**References**

1. Lin SY, Chou AH, Tsai YF, Chang SW, Yang MW, Ting PC, Chen CY. Evaluation of the use of the fourth version FloTrac system in cardiac output measurement before and after cardiopulmonary bypass. J Clin Monit Comput. 2018;32:807-15.

2. Chatti R, de Rudniki S, Marqué S, Dumenil AS, Descorps-Declère A, Cariou A, Duranteau J, Aout M, Vicaut E, Cholley BP. Comparison of two versions of the Vigileo-FloTrac system (1.03 and 1.07) for stroke volume estimation: a multicentre, blinded comparison with oesophageal Doppler measurements. Br J Anaesth. 2009;102:463-9.

3. Thomas ME, Blaine C, Dawnay A, Devonald MA, Ftouh S, Laing C, Latchem S, Lewington A, Milford DV, Ostermann M. The definition of acute kidney injury and its use in practice. Kidney Int. 2015;87:62-73.

4. Shin SR, Kim WH, Kim DJ, Shin IW, Sohn JT. Prediction and Prevention of Acute Kidney Injury after Cardiac Surgery. Biomed Res Int. 2016;2016:2985148.

5. Durand F, Francoz C, Asrani SK, Khemichian S, Pham TA, Sung RS, Genyk YS, Nadim MK. Acute Kidney Injury After Liver Transplantation. Transplantation. 2018;102:1636-49.

6. Olthoff KM, Kulik L, Samstein B, Kaminski M, Abecassis M, Emond J, Shaked A, Christie JD. Validation of a current definition of early allograft dysfunction in liver transplant recipients and analysis of risk factors. Liver Transpl. 2010;16:943-9.

7. Schulte PJ, Mascha EJ. Propensity Score Methods: Theory and Practice for Anesthesia Research. Anesth Analg. 2018;127:1074-84.

8. Durand F, Francoz C, Asrani SK, Khemichian S, Pham TA, Sung RS, Genyk YS, Nadim MK. Acute Kidney Injury After Liver Transplantation. Transplantation. 2018;102:1636-49.

9. Park MH, Shim HS, Kim WH, Kim HJ, Kim DJ, Lee SH, Kim CS, Gwak MS, Kim GS. Clinical Risk Scoring Models for Prediction of Acute Kidney Injury after Living Donor Liver Transplantation: A Retrospective Observational Study. PLoS One. 2015;10:e0136230.

**Supplemental Table S1**. Comparison of patient characteristics and perioperative parameters between the group with and without pulmonary artery catheter (PAC) after matching.

| Characteristic | PAC group  (n= 223) | Non-PAC group  (n= 223) | P-value | Standardized  difference |
| --- | --- | --- | --- | --- |
| Demographic data |  |  |  |  |
| Age, years | 55 (48 – 63) | 55 (49 – 61) | 0.751 | 0.06 |
| Female, n | 71 (31.8) | 71 (31.8) | 0.999 | <0.01 |
| Body-mass index, kg/m^2^ | 23.1 (20.8 – 25.2) | 23.1 (21.2 – 25.6) | 0.669 | 0.04 |
| Background medical status |  |  |  |  |
| Hypertension, n | 24 (10.8) | 24 (10.8) | 0.999 | <0.01 |
| Diabetes mellitus, n | 45 (20.2) | 45 (20.2) | 0.999 | <0.01 |
| Alcoholic liver cirrhosis, n | 19 (8.5) | 23 (10.3) | 0.517 | 0.06 |
| HBV hepatitis, n | 91 (40.8) | 95 (42.6) | 0.701 | 0.04 |
| HCV hepatitis, n | 19 (8.5) | 121 (9.4) | 0.740 | 0.03 |
| Hepatocellular carcinoma, n | 86 (38.6) | 85 (38.1) | 0.922 | 0.01 |
| Cholestatic disease, n | 9 (4.0) | 10 (4.5) | 0.815 | 0.02 |
| Preoperative hemoglobin, g/dl | 8.9 (7.8 – 10.6) | 8.8 (7.7 – 10.2) | 0.756 | 0.01 |
| Preoperative serum albumin level, mg/dl | 3.0 (2.6 – 3.5) | 3.0 (2.5 – 3.4) | 0.638 | <0.01 |
| MELD score | 15 (12 – 21) | 15 (11 – 22) | 0.601 | 0.08 |
| CTP score | 8 (6 – 10) | 8 (6 – 10) | 0.672 | 0.04 |
| Child class, n (A/ B/ C) | 79 (35.4)/ 82 (36.8)/ 62 (27.8) | 85 (38.1)/ 70 (31.4)/ 68 (30.5) | 0.486 | <0.01 |
| Previous abdominal surgery, n | 9 (4.0) | 9 (4.0) | 0.999 | <0.01 |
| Preoperative LVEF, % | 66 (63 – 69) | 66 (63 – 69) | 0.703 | 0.06 |
| Portopulmonary hypertension, n | - | - | - | - |
| Preoperative beta-blocker, n | 9 (4.0) | 9 (4.0) | 0.999 | <0.01 |
| Preoperative diuretics, n | 8 (3.6) | 10 (4.5) | 0.630 | 0.05 |
| Donor/ graft factors |  |  |  |  |
| Deceased/ living, n | 157 (70.4)/ 66 (29.6) | 149 (66.8)/ 74 (33.2) | 0.414 | 0.08 |
| ABO-incompatible transplantation, n | 32 (14.3) | 37 (16.6) | 0.513 | 0.06 |
| Estimated GRWR | 1.20 (1.08 – 1.44) | 1.22 (1.05 – 1.45) | 0.741 | 0.01 |
| Operation and anesthesia details |  |  |  |  |
| Year of transplant |  |  | 0.129 | 0.08 |
| 2005-2010 | 21 (9.4) | 11 (4.9) |  |  |
| 2011-2015 | 49 (22.0) | 44 (19.7) |  |  |
| 2016-2022 | 153 (68.6) | 168 (75.3) |  |  |
| Operation time, hour | 6.6 (5.8 – 8.0) | 6.5 (5.7 – 7.8) | 0.113 | 0.18 |
| Cold ischemic time, min | 89 (68 – 230) | 88 (63 – 240) | 0.798 | 0.06 |
| Warm ischemic time, min | 30 (25 – 34) | 30 (27 – 34) | 0.544 | 0.01 |
| Intraoperative mean blood glucose, mg/dl | 162 (144 – 180) | 162 (144 – 179) | 0.485 | 0.10 |
| Intraoperative crystalloid administration, ml | 3350 (2400 – 5000) | 3500 (2300 – 5000) | 0.933 | 0.07 |
| Intraoperative hydroxyethyl starch administration, ml | 0 (0 – 500) | 0 (0 – 500) | 0.669 | 0.04 |
| 5% albumin, ml | 0 (0 – 0) | 0 (0 – 0) | 0.560 | 0.08 |
| 20% albumin, ml | 500 (0 – 300) | 500 (0 – 300) | 0.336 | 0.04 |
| Bleeding and transfusion amount |  |  |  |  |
| pRBC transfusion, units | 6 (1 – 10) | 6 (2 – 10) | 0.545 | 0.05 |
| FFP transfusion, units | 4 (0 – 11) | 5 (0 – 10) | 0.588 | 0.09 |
| Platelet concentrate, units | 0 (0 – 6) | 0 (0 – 6) | 0.309 | 0.04 |
| Blood loss per body weight, ml/kg | 46 (24 – 92) | 42 (22 – 88) | 0.213 | 0.04 |

The values are expressed as the median [interquartile range] or number (%).

CTP score = Child-Turcotte-Pugh score, LVEF = left ventricular ejection fraction, GRWR = graft versus recipient body weight ratio, p-RBC = packed red blood cells, FFP = fresh frozen plasma.

**Supplemental Table S2**. Comparison of patient characteristics and perioperative parameters between the group with and without pulmonary artery catheter (PAC) in the inverse probability of treatment-weighted cohort.

| Characteristic | PAC group  (n= 786) | Non-PAC group  (n= 779) | P-value | Standardized  difference |
| --- | --- | --- | --- | --- |
| Demographic data |  |  |  |  |
| Age, years | 55 (48 – 62) | 55 (49 – 60) | 0.543 | 0.06 |
| Female, n | 256 (32.6) | 252 (32.3) | 0.926 | <0.01 |
| Body-mass index, kg/m^2^ | 23.1 (20.8 – 25.2) | 23.1 (21.2 – 25.6) | 0.473 | 0.03 |
| Background medical status |  |  |  |  |
| Hypertension, n | 85 (10.8) | 88 (11.3) | 0.761 | 0.02 |
| Diabetes mellitus, n | 157 (20.0) | 156 (20.0) | 0.980 | <0.01 |
| Alcoholic liver cirrhosis, n | 68 (8.7) | 81 (10.4) | 0.239 | 0.06 |
| HBV hepatitis, n | 318 (40.5) | 333 (42.7) | 0.358 | 0.05 |
| HCV hepatitis, n | 65 (8.3) | 78 (10.0) | 0.231 | 0.06 |
| Hepatocellular carcinoma, n | 299 (38.0) | 297 (38.1) | 0.972 | <0.01 |
| Cholestatic disease, n | 33 (4.2) | 36 (4.6) | 0.684 | 0.02 |
| Preoperative hemoglobin, g/dl | 10.6 (8.9 – 12.0) | 10.2 (8.8 – 12.1) | 0.364 | 0.03 |
| Preoperative serum albumin level, mg/dl | 3.0 (2.6 – 3.5) | 3.0 (2.5 – 3.4) | 0.231 | 0.02 |
| MELD score | 15.4 (12.0 – 21.7) | 15.0 (11.0 – 22.3) | 0.229 | 0.07 |
| CTP score | 8 (6 – 10) | 8 (6 – 10) | 0.394 | 0.04 |
| Child class, n (A/ B/ C) | 279 (35.5)/ 293 (37.3)/ 214 (27.2) | 299 (38.4)/ 244 (31.3)/ 236 (30.3) | 0.964 | <0.01 |
| Previous abdominal surgery, n | 30 (3.8) | 30 (3.9) | 0.972 | <0.01 |
| Preoperative LVEF, % | 63 (59 – 66) | 63 (59 – 65) | 0.338 | 0.07 |
| Portopulmonary hypertension, n | - | - | - |  |
| Preoperative beta-blocker, n | 31 (3.9) | 32 (4.1) | 0.869 | <0.01 |
| Preoperative diuretics, n | 30 (3.8) | 37 (4.7) | 0.362 | 0.05 |
| Donor/ graft factors |  |  |  |  |
| Deceased/ living, n | 231 (29.4)/ 555 (70.6) | 258 (33.1)/ 521 (66.9) | 0.111 | 0.08 |
| ABO-incompatible transplantation, n | 99 (12.6) | 120 (15.4) | 0.109 | 0.08 |
| Estimated GRWR | 1.20 (1.08 – 1.45) | 1.23 (1.05 – 1.46) | 0.492 | 0.01 |
| Operation and anesthesia details |  |  |  |  |
| Year of transplant |  |  | 0.859 | 0.02 |
| 2005-2010 | 84 (10.7) | 81 (10.4) |  |  |
| 2011-2015 | 196 (24.9) | 194 (24.9) |  |  |
| 2016-2022 | 506 (64.4) | 504 (64.7) |  |  |
| Operation time, hour | 7.2 (5.7 – 8.4) | 7.1 (5.6 – 8.3) | 0.517 | 0.09 |
| Cold ischemic time, min | 90 (68 – 230) | 88 (63 – 240) | 0.543 | 0.05 |
| Warm ischemic time, min | 30 (25 – 35) | 30 (27 – 34) | 0.248 | 0.02 |
| Intraoperative mean blood glucose, mg/dl | 162 (145 – 179) | 162 (144 – 177) | 0.152 | 0.13 |
| Intraoperative crystalloid administration, ml | 3350 (2400 -5000) | 3500 (2300 – 5000) | 0.894 | 0.09 |
| Intraoperative hydroxyethyl starch administration, ml | 0 (0 – 500) | 0 (0 – 500) | 0.335 | 0.04 |
| 5% albumin, ml | 0 (0 – 0) | 0 (0 – 0) | 0.159 | 0.09 |
| 20% albumin, ml | 200 (0 – 300) | 200 (0 – 300) | 0.237 | 0.05 |
| Bleeding and transfusion amount |  |  |  |  |
| pRBC transfusion, units | 6 (2 – 10) | 6 (2 – 10) | 0.636 | 0.09 |
| FFP transfusion, units | 4 (0 – 11) | 4 (0 – 10) | 0.777 | 0.06 |
| Platelet concentrate, units | 0 (0 – 6) | 0 (0 – 6) | 0.105 | 0.01 |
| Blood loss per body weight, ml/kg | 44 (24 – 93) | 43 (23 – 90) | 0.341 | 0.03 |

The values are expressed as the median [interquartile range] or number (%).

CTP score = Child-Turcotte-Pugh score, LVEF = left ventricular ejection fraction, GRWR = graft versus recipient body weight ratio, p-RBC = packed red blood cells, FFP = fresh frozen plasma.

**Supplemental Table S3**. Comparison of patient characteristics and perioperative parameters between the group with and without pulmonary artery catheter (PAC) in the subgroup of the patients who underwent surgery after the year 2016.

| Characteristic | PAC group  (n= 504) | Non-PAC group  (n= 171) | P-value | Standardized  difference |
| --- | --- | --- | --- | --- |
| Demographic data |  |  |  |  |
| Age, years | 57 (50 – 63) | 56 (51 – 61) | 0.747 | 0.04 |
| Female, n | 142 (28.2) | 54 (31.6) | 0.397 | 0.07 |
| Body-mass index, kg/m^2^ | 23 (21 – 25) | 23 (21 – 26) | 0.486 | 0.09 |
| Background medical status |  |  |  |  |
| Hypertension, n | 62 (12.3) | 13 (7.6) | 0.091 | 0.16 |
| Diabetes mellitus, n | 100 (19.8) | 33 (19.3) | 0.877 | 0.01 |
| Alcoholic liver cirrhosis, n | 61 (12.1) | 19 (11.1) | 0.729 | 0.03 |
| HBV hepatitis, n | 227 (45.0) | 76 (44.4) | 0.892 | 0.01 |
| HCV hepatitis, n | 45 (8.9) | 13 (7.6) | 0.593 | 0.05 |
| Hepatocellular carcinoma, n | 237 (47.0) | 66 (38.6) | 0.056 | 0.17 |
| Cholestatic disease, n | 27 (5.4) | 7 (4.1) | 0.514 | 0.06 |
| Preoperative hemoglobin, g/dl | 11.0 (9.3 – 12.5) | 10.2 (8.8 – 12.4) | 0.101 | 0.13 |
| Preoperative serum albumin level, mg/dl | 3.1 (2.6 – 3.5) | 3.0 (2.5 – 3.4) | 0.113 | 0.11 |
| MELD score | 15.0 (10.8 – 20.8) | 15.7 (11.0 – 22.3) | 0.366 | 0.01 |
| CTP score | 8 (6 – 10) | 8 (6 – 10) | 0.064 | 0.17 |
| Child class, n (A/ B/ C) | 216 (42.9)/ 164 (32.5)/ 124 (24.6) | 64 (37.4)/ 52 (30.4)/ 55 (32.2) | 0.147 | 0.16 |
| Previous abdominal surgery, n | 18 (3.6) | 8 (4.7) | 0.516 | 0.06 |
| Preoperative LVEF, % | 65 (63 – 69) | 65 (63 – 69) | 0.667 | 0.04 |
| Portopulmonary hypertension, n | 0 | 0 | - | - |
| Preoperative beta-blocker, n | 18 (3.6) | 6 (3.5) | 0.970 | 0.00 |
| Preoperative diuretics, n | 29 (5.8) | 8 (4.7) | 0.593 | 0.05 |
| Donor/ graft factors |  |  |  |  |
| Deceased/ living, n | 130 (25.8)/ 374 (74.2) | 51 (29.8)/ 120 (70.2) | 0.304 | 0.09 |
| ABO-incompatible transplantation, n | 91 (18.1) | 39 (22.8) | 0.173 | 0.12 |
| Estimated GRWR | 1.20 (1.06 – 1.42) | 1.22 (1.06 – 1.45) | 0.241 | 0.09 |
| Operation and anesthesia details |  |  |  |  |
| Operation time, hour | 6.7 (5.3 – 7.8) | 6.6 (5.7 – 7.8) | 0.549 | 0.07 |
| Cold ischemic time, min | 93 (69 – 230) | 82 (63 – 240) | 0.276 | 0.02 |
| Warm ischemic time, min | 30 (24 – 34) | 30 (27 – 34) | 0.235 | 0.08 |
| Intraoperative mean blood glucose, mg/dl | 160 (149 – 174) | 162 (149 – 175) | 0.561 | 0.04 |
| Intraoperative crystalloid administration, ml | 3400 (2400 – 5000) | 3500 (2350 – 5000) | 0.631 | 0.02 |
| Intraoperative hydroxyethyl starch administration, ml | 0 (0 – 280) | 0 (0 – 300) | 0.653 | 0.07 |
| 5% albumin, ml | 0 (0 – 0) | 0 (0 – 0) | 0.012 | 0.10 |
| 20% albumin, ml | 200 (100 – 400) | 200 (0 – 400) | 0.147 | 0.14 |
| Bleeding and transfusion amount |  |  |  |  |
| pRBC transfusion, units | 4 (1 – 10) | 6 (2 – 10) | 0.042 | 0.14 |
| FFP transfusion, units | 3 (0 – 8) | 4 (0 – 10) | 0.054 | 0.16 |
| Platelet concentrate, units | 0 (0 – 6) | 0 (0 – 6) | 0.039 | 0.14 |
| Blood loss per body weight, ml/kg | 42.5 (19.6 – 87.7) | 37.7 (20.0 – 88.7) | 0.815 | 0.12 |

The values are expressed as the median [interquartile range] or number (%).

CTP score = Child-Turcotte-Pugh score, LVEF = left ventricular ejection fraction, GRWR = graft versus recipient body weight ratio, p-RBC = packed red blood cells, FFP = fresh frozen plasma.

**Supplemental Table S4**. Comparisons of secondary clinical outcomes after liver transplantation between the PAC and non-PAC group before and after propensity score matching in the subgroup of the patients who underwent surgery after the year 2016.

|  | Before propensity score matching | | | After propensity score matching | | |
| --- | --- | --- | --- | --- | --- | --- |
|  | PAC group  (n= 504) | Non-PAC group  (n= 171) | P-value | PAC group  (n = 166) | Non-PAC group  (n = 166) | P-value |
| Intraoperative events |  |  |  |  |  |  |
| Transfusion ≥ 10 units of pRBC | 80 (15.9) | 26 (15.0) | 0.836 | 28 (16.6) | 25 (14.8) | 0.653 |
| Postreperfusion syndrome, n | 123 (24.5) | 45 (26.1) | 0.617 | 38 (22.9) | 42 (25.6) | 0.608 |
| Epinephrine infusion > 0.05 μg/kg/min | 12 (2.4) | 5 (2.7) | 0.695 | 4 (2.7) | 4 (2.2) | 0.999 |
| Norepinephrine infusion > 0.20 μg/kg/min | 43 (8.6) | 14 (8.4) | 0.889 | 15 (9.0) | 14 (8.5) | 0.846 |
| Postoperative outcomes |  |  |  |  |  |  |
| Acute kidney injury, n |  |  | 0.763 |  |  | 0.362 |
| Stage 1, n | 135 (26.8) | 48 (28.1) |  | 43 (25.9) | 47 (28.3) |  |
| Stage 2, n | 45 (8.9) | 14 (8.2) |  | 14 (8.4) | 14 (8.4) |  |
| Stage 3, n | 21 (4.2) | 6 (3.5) |  | 9 (2.7) | 3 (1.8) |  |
| Acute kidney injury, all stage, n | 201 (39.9) | 68 (39.8) | 0.979 | 66 (39.8) | 64 (38.6) | 0.822 |
| Postoperative hemodialysis, n | 38 (7.5) | 13 (7.6) | 0.979 | 12 (7.2) | 10 (6.0) | 0.659 |
| Postoperative bleeding, n | 20 (4.0) | 5 (2.9) | 0.532 | 4 (2.4) | 5 (3.0) | 0.735 |
| Postoperative wound infection, n | 14 (2.8) | 3 (1.8) | 0.461 | 5 (3.0) | 3 (1.8) | 0.474 |
| Early allograft dysfunction, n | 6 (1.2) | 4 (2.3) | 0.283 | 1 (0.6) | 3 (1.8) | 0.314 |
| In-hospital mortality, n | 10 (2.0) | 2 (1.2) | 0.486 | 2 (1.2) | 2 (1.2) | 0.999 |
| One-year mortality, n | 20 (4.0) | 5 (2.9) | 0.532 | 5 (3.0) | 5 (3.0) | 0.999 |
| Length of ICU stay, days | 5 (4 – 7) | 5 (4 – 8) | 0.215 | 7 (5 – 11) | 7 (5 – 12) | 0.537 |
| Length of hospital stay, days | 18 (15 – 27) | 19 (15 – 30) | 0.197 | 19 (14 – 28) | 19 (15 – 29) | 0.474 |

Data are presented as the number (%) or median [interquartile range] or number (%). ICU = intensive care unit.

Postreperfusion syndrome was defined as a >30% decrease of mean blood pressure at least 1 minute within 5 minutes of portal vein reperfusion compared with the baseline observed immediately before reperfusion.

**Supplemental Table S5**. Results of ordinal logistic regression analysis for early allograft dysfunction and acute kidney injury after liver transplantation using inverse probability of treatment weighting in the subgroup of the patients who underwent surgery after the year 2016.

|  | Logistic regression analysis  (n = 718) | |
| --- | --- | --- |
|  | Odds ratio (95% CI) | P-value |
| PAC group vs. non-PAC group  (For acute kidney injury) | 1.16 (0.37 – 1.85) | 0.317 |
| PAC group vs. non-PAC group  (For early allograft dysfunction) | 0.90 (0.44 – 1.66) | 0.754 |

PAC = pulmonary artery catheter, CI = confidence interval.

This analysis was performed in the inverse probability of treatment-weighted cohort (n= 718).

**Supplemental Table S6**. Subgroup analysis of the highest quartile of model for end-stage liver disease score (≥ 22) for the comparisons of intraoperative event and secondary clinical outcomes after liver transplantation between the PAC and non-PAC group before and after propensity score matching (n=398).

|  | Before propensity score matching | | | After propensity score matching | | |
| --- | --- | --- | --- | --- | --- | --- |
|  | PAC group  (n= 338) | Non-PAC group  (n= 60) | P-value | PAC group  (n = 36) | Non-PAC group  (n = 36) | P-value |
| Intraoperative events |  |  |  |  |  |  |
| Transfusion ≥ 10 units of pRBC | 51 (15.1) | 10 (16.7) | 0.755 | 5 (13.9) | 7 (19.4) | 0.527 |
| Postreperfusion syndrome, n | 80 (23.7) | 21 (35.0) | 0.063 | 8 (22.2) | 16 (44.4) | 0.046 |
| Epinephrine infusion > 0.05 μg/kg/min | 8 (2.4) | 1 (1.7) | 0.737 | 1 (2.8) | 3 (8.3) | 0.303 |
| Norepinephrine infusion > 0.20 μg/kg/min | 28 (8.3) | 5 (8.3) | 0.990 | 2 (5.6) | 4 (11.1) | 0.394 |
| Postoperative outcomes |  |  |  |  |  |  |
| Acute kidney injury, n |  |  |  |  |  |  |
| Stage 1, n | 95 (28.1) | 14 (23.3) | 0.445 | 8 (22.2) | 11 (30.6) | 0.422 |
| Stage 2, n | 24 (7.1) | 6 (10.0) | 0.433 | 3 (8.3) | 4 (11.1) | 0.691 |
| Stage 3, n | 10 (3.0) | 3 (5.0) | 0.412 | 1 (2.8) | 2 (5.6) | 0.555 |
| Acute kidney injury, all stage, n | 129 (38.2) | 23 (38.3) | 0.980 | 12 (33.3) | 17 (47.2) | 0.230 |
| Postoperative hemodialysis, n | 23 (6.8) | 5 (8.3) | 0.670 | 2 (5.6) | 4 (11.1) | 0.394 |
| Postoperative bleeding, n | 15 (4.4) | 2 (3.3) | 0.697 | 2 (5.6) | 2 (5.6) | 0.999 |
| Postoperative wound infection, n | 6 (1.8) | 1 (1.7) | 0.953 | 1 (2.8) | - | 0.314 |
| Early allograft dysfunction, n | 10 (3.0) | 2 (3.3) | 0.876 | 1 (2.8) | 1 (2.8) | 0.999 |
| In-hospital mortality, n | 2 (0.6) | 1 (1.7) | 0.375 | 1 (2.8) | - | 0.314 |
| One-year mortality, n | 16 (4.7) | 2 (3.3) | 0.630 | 2 (5.6) | 2 (5.6) | 0.999 |
| Length of ICU stay, days | 5 (4 – 8) | 5 (4 – 7) | 0.349 | 5 (4 – 7) | 6 (4 – 9) | 0.428 |
| Length of hospital stay, days | 18 (14 – 26) | 19 (15 – 28) | 0.248 | 17 (14 – 28) | 19 (16 – 29) | 0.191 |

Data are presented as the number (%) or median [interquartile range] or number (%). ICU = intensive care unit, pRBC = packed red blood cell.

Postreperfusion syndrome was defined as a >30% decrease of mean blood pressure at least 1 minute within 5 minutes of portal vein reperfusion compared with the baseline observed immediately before reperfusion.

**Supplemental Table S7**. Subgroup analysis of the low three quartiles of model for end-stage liver disease score (< 22) for the comparisons of intraoperative event and secondary clinical outcomes after liver transplantation between the PAC and non-PAC group before and after propensity score matching (n = 1167).

|  | Before propensity score matching | | | After propensity score matching | | |
| --- | --- | --- | --- | --- | --- | --- |
|  | PAC group  (n= 1001) | Non-PAC group  (n= 166) | P-value | PAC group  (n = 135) | Non-PAC group  (n = 135) | P-value |
| Intraoperative events |  |  |  |  |  |  |
| Transfusion ≥ 10 units of pRBC | 145 (14.5) | 25 (15.1) | 0.846 | 20 (14.8) | 22 (16.3) | 0.737 |
| Postreperfusion syndrome, n | 238 (23.8) | 37 (22.3) | 0.676 | 34 (25.2) | 31 (23.0) | 0.669 |
| Epinephrine infusion > 0.05 μg/kg/min | 24 (2.4) | 4 (2.4) | 0.993 | 3 (2.2) | 3 (2.2) | 0.999 |
| Norepinephrine infusion > 0.20 μg/kg/min | 79 (7.9) | 14 (8.4) | 0.811 | 11 (8.1) | 10 (7.4) | 0.820 |
| Postoperative outcomes |  |  |  |  |  |  |
| Acute kidney injury, n |  |  |  |  |  |  |
| Stage 1, n | 327 (32.7) | 43 (25.9) | 0.083 | 38 (28.1) | 35 (25.9) | 0.681 |
| Stage 2, n | 73 (7.3) | 12 (7.2) | 0.977 | 9 (6.7) | 10 (7.4) | 0.812 |
| Stage 3, n | 50 (5.0) | 7 (4.2) | 0.667 | 5 (3.7) | 6 (4.4) | 0.758 |
| Acute kidney injury, all stage, n | 450 (45.0) | 62 (37.3) | 0.067 | 52 (38.5) | 51 (37.8) | 0.900 |
| Postoperative hemodialysis, n | 68 (6.8) | 12 (7.2) | 0.837 | 8 (5.9) | 10 (7.4) | 0.626 |
| Postoperative bleeding, n | 43 (4.3) | 7 (4.2) | 0.963 | 8 (5.9) | 5 (3.7) | 0.394 |
| Postoperative wound infection, n | 33 (3.3) | 2 (1.2) | 0.143 | 4 (3.0) | 2 (1.5) | 0.409 |
| Early allograft dysfunction, n | 28 (2.8) | 5 (3.0) | 0.877 | 5 (3.7) | 4 (3.0) | 0.735 |
| In-hospital mortality, n | 15 (1.5) | 1 (0.6) | 0.358 | 2 (1.5) | 1 (0.7) | 0.562 |
| One-year mortality, n | 48 (4.8) | 7 (4.2) | 0.745 | 6 (4.4) | 4 (4.4) | 0.999 |
| Length of ICU stay, days | 5 (3 – 7) | 5 (4 – 8) | 0.248 | 5 (4 – 8) | 5 (4 – 7) | 0.541 |
| Length of hospital stay, days | 19 (16 – 28) | 18 (15 – 29) | 0.941 | 18 (15 – 29) | 19 (16 – 28) | 0.425 |

Data are presented as the number (%) or median [interquartile range] or number (%). ICU = intensive care unit, pRBC = packed red blood cell.

Postreperfusion syndrome was defined as a >30% decrease of mean blood pressure at least 1 minute within 5 minutes of portal vein reperfusion compared with the baseline observed immediately before reperfusion.

**Supplemental Table S8**. Subgroup analysis of the highest quartile of cold ischemic time (≥ 240 min) for the comparisons of intraoperative event and secondary clinical outcomes after liver transplantation between the PAC and non-PAC group before and after propensity score matching (n = 454).

|  | Before propensity score matching | | | After propensity score matching | | |
| --- | --- | --- | --- | --- | --- | --- |
|  | PAC group  (n= 379) | Non-PAC group  (n= 75) | P-value | PAC group  (n = 59) | Non-PAC group  (n = 59) | P-value |
| Intraoperative events |  |  |  |  |  |  |
| Transfusion ≥ 10 units of pRBC | 53 (14.0) | 12 (16.0) | 0.649 | 7 (11.9) | 10 (16.9) | 0.432 |
| Postreperfusion syndrome, n | 90 (23.7) | 19 (25.3) | 0.769 | 8 (13.6) | 18 (30.5) | 0.026 |
| Epinephrine infusion > 0.05 μg/kg/min | 9 (2.4) | 2 (2.7) | 0.881 | - | 2 (3.4) | 0.154 |
| Norepinephrine infusion > 0.20 μg/kg/min | 32 (8.4) | 6 (8.0) | 0.899 | 3 (5.1) | 6 (10.2) | 0.298 |
| Postoperative outcomes |  |  |  |  |  |  |
| Acute kidney injury, n |  |  |  |  |  |  |
| Stage 1, n | 120 (31.7) | 19 (25.3) | 0.277 | 11 (18.6) | 18 (30.5) | 0.134 |
| Stage 2, n | 28 (7.4) | 6 (8.0) | 0.854 | 3 (5.1) | 6 (10.2) | 0.298 |
| Stage 3, n | 17 (4.5) | 3 (4.0) | 0.852 | - | 3 (5.1) | 0.079 |
| Acute kidney injury, all stage, n | 165 (43.5) | 28 (37.3) | 0.321 | 14 (23.7) | 27 (45.8) | 0.012 |
| Postoperative hemodialysis, n | 26 (6.9) | 6 (8.0) | 0.725 | 3 (5.1) | 4 (6.8) | 0.697 |
| Postoperative bleeding, n | 16 (4.2) | 4 (5.3) | 0.668 | 4 (6.8) | 2 (3.4) | 0.402 |
| Postoperative wound infection, n | 11 (2.9) | 1 (1.3) | 0.439 | 2 (3.4) | 1 (1.7) | 0.559 |
| Early allograft dysfunction, n | 11 (2.9) | 3 (4.0) | 0.615 | 2 (3.4) | 3 (5.1) | 0.648 |
| In-hospital mortality, n | 6 (1.6) | 1 (1.3) | 0.873 | 1 (1.7) | 1 (1.7) | 0.999 |
| One-year mortality, n | 18 (4.7) | 4 (5.3) | 0.830 | 3 (5.1) | 4 (6.8) | 0.697 |
| Length of ICU stay, days |  |  |  |  |  |  |
| Length of hospital stay, days |  |  |  |  |  |  |

Data are presented as the number (%) or median [interquartile range] or number (%). ICU = intensive care unit, pRBC = packed red blood cell.

Postreperfusion syndrome was defined as a >30% decrease of mean blood pressure at least 1 minute within 5 minutes of portal vein reperfusion compared with the baseline observed immediately before reperfusion.

**Supplemental Table S9**. Subgroup analysis of the low three quartiles of cold ischemic time (< 240 min) for the comparisons of intraoperative event and secondary clinical outcomes after liver transplantation between the PAC and non-PAC group before and after propensity score matching (n = 1111).

|  | Before propensity score matching | | | After propensity score matching | | |
| --- | --- | --- | --- | --- | --- | --- |
|  | PAC group  (n= 960) | Non-PAC group  (n= 151) | P-value | PAC group  (n = 112) | Non-PAC group  (n = 112) | P-value |
| Intraoperative events |  |  |  |  |  |  |
| Transfusion ≥ 10 units of pRBC | 149 (15.5) | 23 (15.2) | 0.927 | 17 (15.2) | 19 (17.0) | 0.716 |
| Postreperfusion syndrome, n | 228 (23.8) | 39 (25.8) | 0.579 | 28 (25.0) | 26 (23.2) | 0.755 |
| Epinephrine infusion > 0.05 μg/kg/min | 23 (2.4) | 3 (2.0) | 0.757 | 2 (1.8) | 3 (2.7) | 0.999 |
| Norepinephrine infusion > 0.20 μg/kg/min | 78 (8.1) | 13 (8.6) | 0.840 | 7 (6.3) | 9 (8.0) | 0.999 |
| Postoperative outcomes |  |  |  |  |  |  |
| Acute kidney injury, n |  |  |  |  |  |  |
| Stage 1, n | 301 (31.4) | 38 (25.2) | 0.125 | 27 (24.1) | 31 (27.7) | 0.542 |
| Stage 2, n | 70 (7.3) | 12 (7.9) | 0.775 | 10 (8.9) | 9 (8.0) | 0.810 |
| Stage 3, n | 43 (4.5) | 7 (4.6) | 0.931 | 5 (4.5) | 5 (4.5) | 0.999 |
| Acute kidney injury, all stage, n | 414 (43.1) | 57 (37.7) | 0.214 | 42 (37.5) | 45 (40.2) | 0.681 |
| Postoperative hemodialysis, n | 65 (6.8) | 11 (7.3) | 0.816 | 7 (6.3) | 8 (7.1) | 0.789 |
| Postoperative bleeding, n | 42 (4.4) | 5 (3.3) | 0.546 | 7 (6.3) | 5 (4.5) | 0.999 |
| Postoperative wound infection, n | 28 (2.9) | 2 (1.3) | 0.262 | 4 (3.6) | 2 (1.8) | 0.408 |
| Early allograft dysfunction, n | 27 (2.8) | 4 (2.6) | 0.910 | 3 (2.7) | 4 (3.6) | 0.701 |
| In-hospital mortality, n | 11 (1.1) | 1 (0.7) | 0.593 | 2 (1.8) | 1 (0.9) | 0.561 |
| One-year mortality, n | 46 (4.8) | 5 (3.3) | 0.419 | 4 (3.6) | 5 (4.5) | 0.734 |
| Length of ICU stay, days |  |  |  |  |  |  |
| Length of hospital stay, days |  |  |  |  |  |  |

Data are presented as the number (%) or median [interquartile range] or number (%). ICU = intensive care unit, pRBC = packed red blood cell.

Postreperfusion syndrome was defined as a >30% decrease of mean blood pressure at least 1 minute within 5 minutes of portal vein reperfusion compared with the baseline observed immediately before reperfusion.

**Supplemental Table S10**. Subgroup analysis of the highest quartile of warm ischemic time (≥ 35 min) for the comparisons of intraoperative event and secondary clinical outcomes after liver transplantation between the PAC and non-PAC group before and after propensity score matching (n = 425).

|  | Before propensity score matching | | | After propensity score matching | | |
| --- | --- | --- | --- | --- | --- | --- |
|  | PAC group  (n= 365) | Non-PAC group  (n= 60) | P-value | PAC group  (n = 38) | Non-PAC group  (n = 38) | P-value |
| Intraoperative events |  |  |  |  |  |  |
| Transfusion ≥ 10 units of pRBC | 60 (16.4) | 9 (15.0) | 0.970 | 7 (18.4) | 7 (18.4) | 0.999 |
| Postreperfusion syndrome, n | 87 (23.8) | 16 (26.7) | 0.423 | 8 (21.1) | 11 (28.9) | 0.427 |
| Epinephrine infusion > 0.05 μg/kg/min | 8 (2.2) | 1 (1.7) | 0.853 | 1 (2.6) | 1 (2.6) | 0.999 |
| Norepinephrine infusion > 0.20 μg/kg/min | 32 (8.8) | 5 (8.3) | 0.951 | 2 (5.3) | 3 (7.9) | 0.644 |
| Postoperative outcomes |  |  |  |  |  |  |
| Acute kidney injury, n |  |  |  |  |  |  |
| Stage 1, n | 126 (34.5) | 17 (28.3) | 0.579 | 9 (23.7) | 11 (28.9) | 0.602 |
| Stage 2, n | 27 (7.4) | 6 (10.0) | 0.379 | 3 (7.9) | 4 (10.5) | 0.692 |
| Stage 3, n | 18 (4.9) | 5 (8.3) | 0.213 | 2 (5.3) | 3 (7.9) | 0.644 |
| Acute kidney injury, all stage, n | 171 (46.8) | 28 (46.7) | 0.623 | 14 (36.8) | 18 (47.4) | 0.353 |
| Postoperative hemodialysis, n | 27 (7.4) | 5 (8.3) | 0.572 | 2 (5.3) | 3 (7.9) | 0.644 |
| Postoperative bleeding, n | 16 (4.4) | 2 (3.3) | 0.791 | 3 (7.9) | 2 (5.3) | 0.644 |
| Postoperative wound infection, n | 11 (3.0) | 1 (1.7) | 0.615 | 2 (5.3) | 1 (2.6) | 0.556 |
| Early allograft dysfunction, n | 12 (3.3) | 2 (3.3) | 0.902 | 1 (2.6) | 2 (5.3) | 0.556 |
| In-hospital mortality, n | 5 (1.4) | 1 (1.7) | 0.800 | 1 (2.6) | 1 (2.6) | 0.999 |
| One-year mortality, n | 14 (3.8) | 2 (3.3) | 0.934 | 1 (2.6) | 2 (5.3) | 0.556 |
| Length of ICU stay, days |  |  |  |  |  |  |
| Length of hospital stay, days |  |  |  |  |  |  |

Data are presented as the number (%) or median [interquartile range] or number (%). ICU = intensive care unit, pRBC = packed red blood cell.

Postreperfusion syndrome was defined as a >30% decrease of mean blood pressure at least 1 minute within 5 minutes of portal vein reperfusion compared with the baseline observed immediately before reperfusion.

**Supplemental Table S11**. Subgroup analysis of the low three quartiles of warm ischemic time (< 35 min) for the comparisons of intraoperative event and secondary clinical outcomes after liver transplantation between the PAC and non-PAC group before and after propensity score matching (n = 1140)

|  | Before propensity score matching | | | After propensity score matching | | |
| --- | --- | --- | --- | --- | --- | --- |
|  | PAC group  (n= 974) | Non-PAC group  (n= 166) | P-value | PAC group  (n = 108) | Non-PAC group  (n = 108) | P-value |
| Intraoperative events |  |  |  |  |  |  |
| Transfusion ≥ 10 units of pRBC | 147 (15.1) | 26 (15.7) | 0.842 | 16 (14.8) | 20 (18.5) | 0.465 |
| Postreperfusion syndrome, n | 232 (23.8) | 40 (24.1) | 0.927 | 27 (25.0) | 25 (23.1) | 0.750 |
| Epinephrine infusion > 0.05 μg/kg/min | 24 (2.5) | 4 (2.4) | 0.952 | 2 (1.9) | 3 (2.8) | 0.651 |
| Norepinephrine infusion > 0.20 μg/kg/min | 80 (8.2) | 14 (8.4) | 0.918 | 9 (8.3) | 8 (7.4) | 0.801 |
| Postoperative outcomes |  |  |  |  |  |  |
| Acute kidney injury, n |  |  |  |  |  |  |
| Stage 1, n | 297 (30.5) | 42 (25.3) | 0.181 | 26 (24.1) | 28 (25.9) | 0.753 |
| Stage 2, n | 69 (7.1) | 9 (5.4) | 0.157 | 10 (9.3) | 9 (8.3) | 0.810 |
| Stage 3, n | 42 (4.3) | 6 (3.6) | 0.683 | 4 (3.7) | 4 (3.7) | 0.999 |
| Acute kidney injury, all stage, n | 408 (41.9) | 57 (34.3) | 0.070 | 40 (37.0) | 41 (38.0) | 0.888 |
| Postoperative hemodialysis, n | 66 (6.8) | 12 (7.2) | 0.826 | 6 (5.6) | 8 (7.4) | 0.580 |
| Postoperative bleeding, n | 44 (4.5) | 7 (4.2) | 0.867 | 6 (5.6) | 4 (3.7) | 0.517 |
| Postoperative wound infection, n | 28 (2.9) | 2 (1.2) | 0.215 | 4 (3.7) | 1 (0.9) | 0.175 |
| Early allograft dysfunction, n | 26 (2.7) | 5 (3.0) | 0.799 | 3 (2.8) | 3 (2.8) | 0.999 |
| In-hospital mortality, n | 12 (1.2) | 1 (0.6) | 0.481 | 2 (1.9) | 1 (0.9) | 0.561 |
| One-year mortality, n | 54 (5.5) | 7 (4.2) | 0.486 | 5 (4.6) | 4 (3.7) | 0.733 |
| Length of ICU stay, days |  |  |  |  |  |  |
| Length of hospital stay, days |  |  |  |  |  |  |

Data are presented as the number (%) or median [interquartile range] or number (%). ICU = intensive care unit, pRBC = packed red blood cell.

Postreperfusion syndrome was defined as a >30% decrease of mean blood pressure at least 1 minute within 5 minutes of portal vein reperfusion compared with the baseline observed immediately before reperfusion.

**Supplemental Figure S1**. Histogram (left) shows the distribution of propensity scores before and after matching. Covariate balance plot (right) compares the standardized differences before and after the propensity score matching.


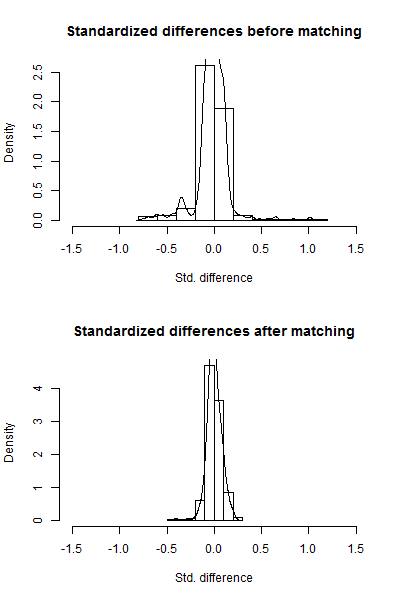

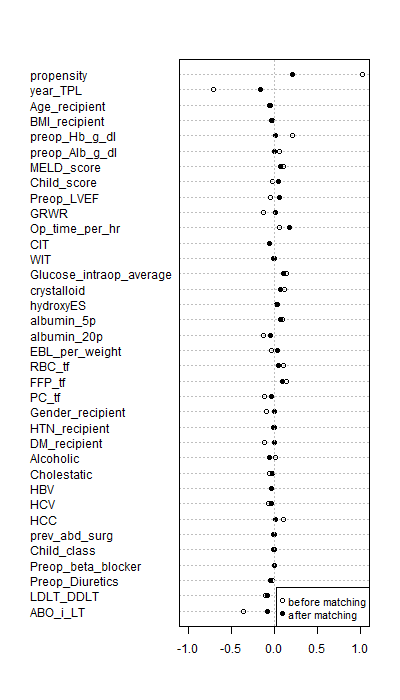


year_TPL = year of liver transplantation, BMI = body-mass index, preop_Hb_g_dl = preoperative serum hemoglobin (g/dL), preop_Alb_g_dl = preoperative serum albumin (g/dL), MELD = model for end-stage liver disease, Preop_LVEF = preoperative left ventricular ejection fraction, GRWR = graft-to-recipient weight ratio, Op_time_per_hr = operation time per hour, CIT = cold ischemic time, WIT = warm ischemic time, Glucose_intraop_average = intraoperative mean blood gluocose level, hydroxyES = intraoperative administration of hydroxy ethyl starch, albumin_5p = intraoperative 5% albumin administration, albumin_20p = intraoperative 20% albumin administration, EBL = estimated blood loss, RBC_tf = intraoperative red blood cell transfusion, FFP_tf = intraoperative fresh frozen plasma transfusion, PC_tf = intraoperative platelet concentrate transfusion, HTN_recipient = history of hypertension, DM_recipient = history of diabetes mellitus, alcoholic = history of alcoholic liver cirrhosis, cholestatic = history of cholestatic liver cirrhosis, HBV = hepatitis B virus, HCV = hepatitis C virus, HCC = hepatocellualr carcinoma, prev_abd_surg = history of previous abdominal surgery, preop_beta_blocker = preoperative administration of beta-blocker, preop_diuretics = preoperative adminstration of diuretics, LDLT_DDLT = living vs. deceased donor case, ABO_i_LT = ABO-incompatible liver transplantation.
